# Schwannoma neuroblastomoide formador de rosetas: presentación de caso con discusión histopatológica y revisión de la literatura

**DOI:** 10.7705/biomedica.7416

**Published:** 2026-06-01

**Authors:** Diana Caicedo, Natalia Rodríguez, Rafael Vélez, Reinhard Rodríguez, Rodrigo Restrepo

**Affiliations:** 1 Sección Dermatopatología, Universidad CES, Medellín, Colombia Universidad CES Sección Dermatopatología Universidad CES Medellín Colombia; 2 Sección Medicina General, Universidad Libre, Barranquilla, Colombia Universidad Libre Sección Medicina General Universidad Libre Barranquilla Colombia; 3 Sección Patología General, Fundación Universitaria de Ciencias de la Salud, Bogotá, D. C., Colombia Fundación Universitaria de Ciencias de la Salud Sección Patología General Fundación Universitaria de Ciencias de la Salud Bogotá, D. C. Colombia; 4 Laboratorio Dr. Rodrigo Restrepo SAS, Medellín, Colombia Laboratorio Dr. Rodrigo Restrepo SAS Laboratorio Dr. Rodrigo Restrepo SAS Medellín Colombia

**Keywords:** neurilemoma, diagnóstico diferencial, inmunohistoquímica, informes de casos, Neurilemmoma, diagnosis, differential, immunohistochemistry, case reports

## Abstract

El schwannoma neuroblastomoide formador de rosetas se considera una variante del schwannoma y representa un reto diagnóstico, dada su baja incidencia y los escasos reportes en la literatura.

Se presenta el caso de una mujer de 40 años con un nodulo doloroso de crecimiento progresivo localizado en la pared anterior del tórax. Después del estudio histopatológico e inmunofenotípico, y de una revisión de los casos reportados en la literatura con énfasis en el diagnóstico diferencial y la inmunohistoquímica, se diagnosticó un schwannoma similar al neuroblastoma y formador de rosetas.

## Abstract (en)

The neuroblastoma-like schwannoma is considered a very rare variant of schwannoma, and represents a diagnostic challenge given its low incidence and few reports.

We present the case of a 40-year-old woman with a painful nodule with progressive growth in the anterior thoracic wall. After histopathological and immunophenotypic analysis, and a literature case review, with emphasis in the differential diagnosis and immunohistochemical analysis, the diagnosis of neuroblastoma-like schwannoma was made.

Los schwannomas son tumores benignos de la vaina nerviosa con múltiples variantes histológicas, entre las que se describe el schwannoma neuroblastomoide formador de rosetas, que presenta características histológicas muy llamativas, además de un diagnóstico diferencial extenso. El hecho de que se trate de una lesión poco común con escasos reportes en la literatura incrementa la importancia de una revisión histopatológica e inmunohistoquímica.

## Caso clínico

Se trata de una paciente de sexo femenino, de 40 años, sin antecedentes clínicos relevantes. Consultó por una sensación de masa en la pared torácica anterior. Clínicamente, se identificó una lesión nodular en el tejido celular subcutáneo, blanda, dolorosa a la palpación, móvil y no adherida a los planos profundos. Se resecó el nodulo y se practicaron los estudios de histopatología e inmunohistoquímica.

**Figura 1.**
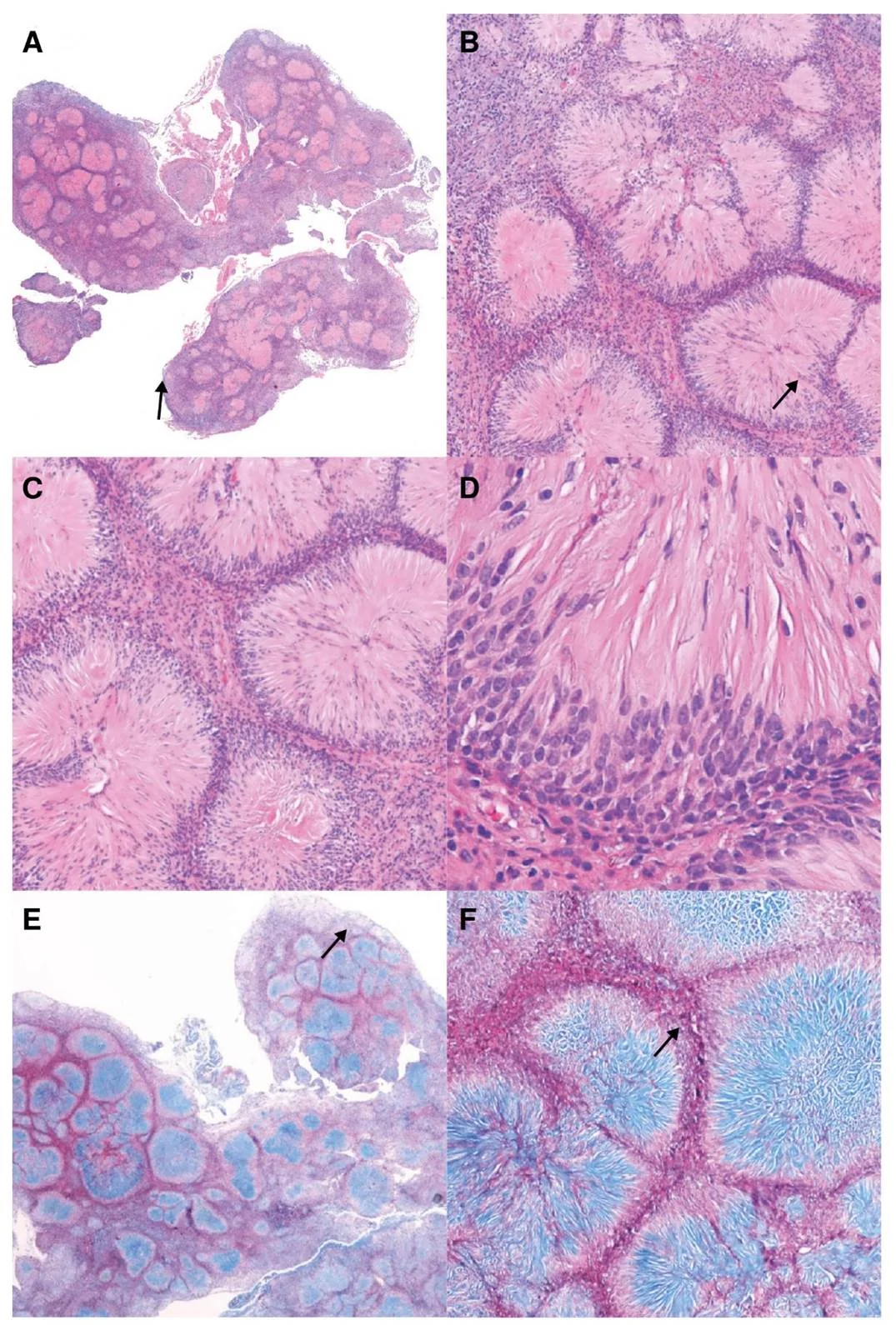
Schwannoma neuroblastomoide formador de rosetas. **A)** Lesión mesenquimatosa de bordes bien delimitados, hematoxilina y eosina, 4X. **B)** Presencia de estructuras rosetoides, hematoxilina y eosina, 10X. **C)** Estructuras rosetoides constituidas por células pequeñas de núcleos hipercromáticos, hematoxilina y eosina, 20X. **D)** Estructuras rosetoides con células dispuestas en la periferia con centro fibrilar colágeno radiado, hematoxilina y eosina, 40X. **E)** Centros resaltados con tinciones como la tricrómica de Masson, 10X. **F)** Tinción con tricrómico de Masson, 40X.

Microscópicamente, se identificó una lesión mesenquimatosa de bordes bien delimitados. Resultó llamativa la presencia de estructuras en forma de rosetas constituidas por células pequeñas de núcleos hipercromáticos dispuestas en la periferia, con centro fibrilar colágeno radiado. En las áreas adyacentes, se observaron células fusiformes pequeñas que recordaban a un schwannoma de patrón convencional. Las tinciones de histoquímica con la tricrómica de Masson resaltaban los centros fibrilares de colágeno (figura 1).

**Figura 2.**
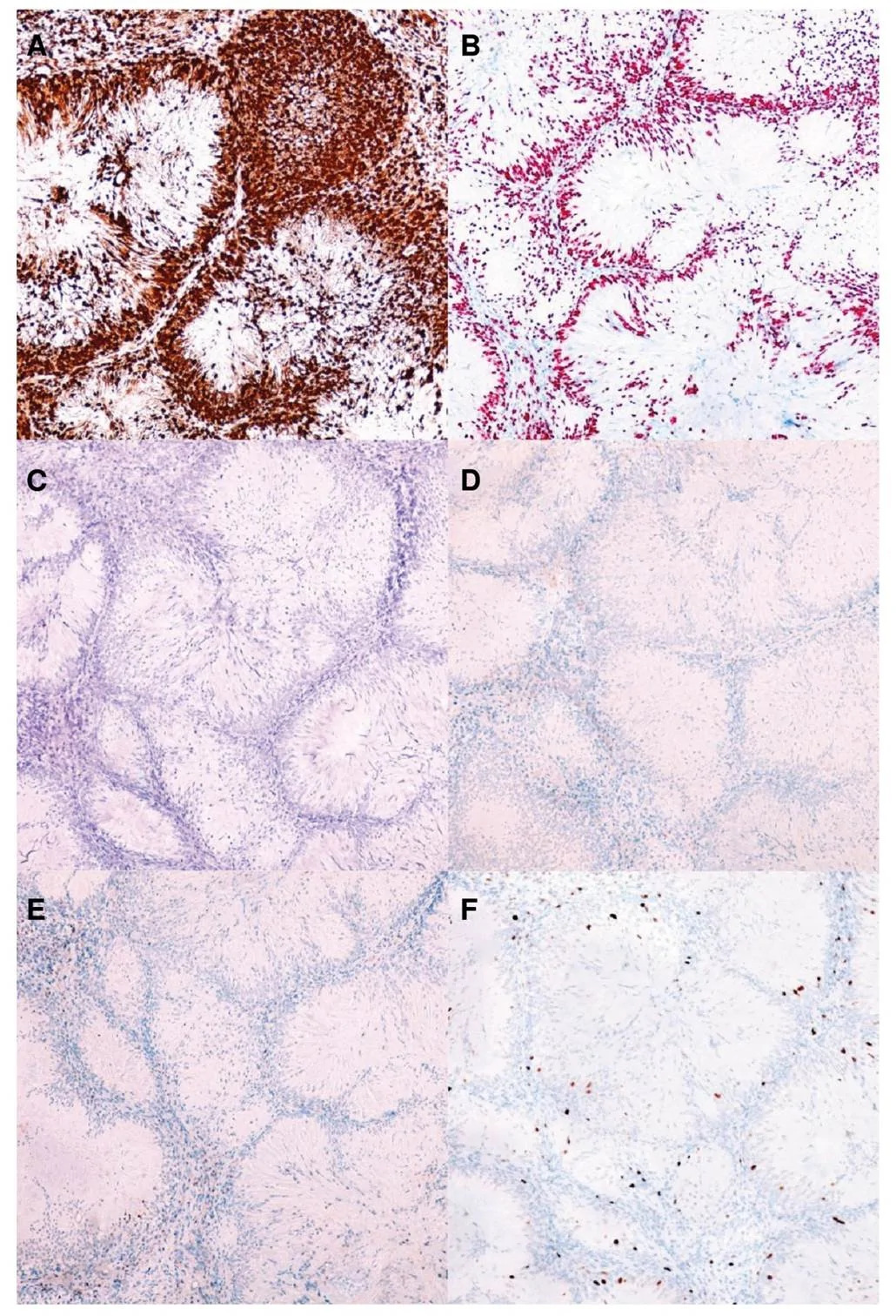
Resumen de expresión inmunohistoquímica: **A)** Positividad fuerte y difusa para S100. **B)** Positividad fuerte y difusa para SOX10. **C)** Negatividad para neurofilamento. **D)** Negatividad para MelanA. **E)** Negatividad para EMA. **F)** Reactividad nuclear para KI67 en un 5 %.

Las tinciones de inmunohistoquímica mostraron positividad fuerte y difusa para S100 (anticuerpo 4C4.9) y SOX10, con un bajo índice de proliferación de Ki-67. Las tinciones para neurofilamento MelanA, EMA, CD99, Desmina, MUC4, MITF1 y AML resultaron negativas con un índice bajo de Ki-67 (figura 2). Los resultados de inmunohistoquímica se resumen en el cuadro 1.

**Cuadro 1.** Resumen de la marcación de inmunohistoquímica en el presente caso

	Positivo	Negativo
CKC		X
EMA		X
AML		X
Desmina		X
H-Caldesmon		X
CD10	Débil	
MUC4		X
S100	Fuerte, difuso	
NF		X
STAT6		X
SOX10	Fuerte, nuclear	
MITF1		X
MelanA		X
CiclinaD1		X
CD99		X
FXIIIa	Débil	
D2-40	Débil	
CD34		X
CD31		X
B-catenina	Débil, citoplasmático	
BCL2		X
Ki67	5%	

Teniendo en cuenta las características histológicas e inmunofenotípicas, se diagnosticó un schwannoma neuroblastomoide formador de rosetas.

## Consideraciones éticas

Según la declaración de Helsinki y las directrices de la 67^a^ *Asamblea General de la Asociación Médica Mundial* sobre las declaraciones éticas de las bases de datos de salud y los biobancos (2016), la presente investigación se ajusta a los principios éticos fundamentales y ha sido aprobada por el Comité de Ética en Investigación de la Universidad CES, garantizando la protección de datos y reserva, buscando minimizar cualquier daño potencial. Además, los posibles conflictos de interés de los autores han sido declarados. De igual manera, el investigador se compromete a mantener en reserva cualquier información obtenida del estudio. En cualquier caso, el investigador principal asumirá la responsabilidad de la preservación de la confidencialidad.

## Discusión

Los schwannomas son tumores benignos de la vaina nerviosa periférica que surgen de las células de Schwann, usualmente encapsulados y bien circunscritos, compuestos por áreas celulares (Antoni A), asociados con una empalizada nuclear (cuerpos de Verocay) y un componente hipocelular (Antoni B) ^1^^-^^4^.

En la clasificación histológica de los schwannomas, se describen algunas variantes, como la epitelioide, la celular y la pigmentada; el caso que nos compete ^2^, el schwannoma neuroblastomoide formador de rosetas ^5^. Esta última variante representa un reto diagnóstico dada su baja incidencia y los escasos reportes en la literatura. Hasta la fecha, hay 26 casos reportados, incluyendo el caso actual, en el cual se resalta la importancia del patrón histológico y los diagnósticos diferenciales.

Esta variante fue descrita inicialmente por Goldblum *et al.* en 1994 ^6^ como una lesión mesenquimatosa con ligero predominio en mujeres jóvenes, ya sea de forma aislada o en relación con schwannomatosis. Clínicamente, se presenta como nodulos subcutáneos, ocasionalmente dolorosos, localizados en las extremidades o en el tronco ^7^.

Entre los 26 casos reportados en la literatura, se encontró que la edad promedio de presentación fue de 34 años, siendo más frecuente en el sexo femenino (77 %). Las localizaciones anatómicas más frecuentes son el flanco, el tórax, la rodilla, el tobillo, el antebrazo y el brazo. El diámetro tumoral promedio fue de 26 mm (cuadro 2).

**Cuadro 2.** Reportes de casos de Schwannoma neuroblastomoide formador de rosetas por año de publicación

Autor, año y referencia	Sexo	Edad (años)	Localización	Tamaño (mm)
Goldblum *et al.,* 1994 ^6^	M	32	Palma	15
Goldblum *et al*., 1994 ^6^	F	44	Cuello	45
Goldblum *et al*., 1994 ^6^	F	46	Flanco	13
Skelton *et al.,* 1994 ^8^	F	41	Brazo	8
Fisher *et al.,* 1995 ^9^	F	24	Muslo	20
Bhatnagar *et al*., 1998 ^10^	F	53	Vulva	20
Bhatnagar *et al*., 1998 ^10^	F	35	Rodilla	18
De Saint Aubain Somerhausen *et al*., 2003 ^11^	SD	62	Cuello	6
Lewis *et al.,* 2005 ^1^	F	40	Tobillo	8
Lewis *et al*., 2005 ^1^	F	24	Rodilla	39
Vélez *et al.,* 2006 ^12^	F	29	Glúteo	SD
Kukreja *et al*., 2007 ^13^	M	16	Órbita	21
Mourmouras *et al.,* 2008 ^14^	F	38	Flanco	40
Suchak *et al.,* 2010 ^2^	F	25	Espalda	SD
Suchak *et al*., 2010 ^2^	F	32	Mama	SD
Sharma *et al*., 2010 ^15^	F	61	Intradural	12
Carter *et al*., 2012 ^16^	F	31	Pierna	67
			Codo	SD
Gutte, 2014^17^	M	26	Labio inferior	8
Tavares *et al.,* 2014 ^18^	M	26	Labio inferior	25
Adams *et al.,* 2015 ^19^	F	45	Pleura	27
Sulhyan *et al.,* 2015 ^20^	F	19	Tobillo	15
Kaur *et al*., 2016 ^21^	F	44	Base del cráneo	88
Geramizadeh *et al*., 2016 ^22^	F	36	Intradural	40
Sharma *et al*., 2017 ^7^	M	21	Antebrazo	15
Mahjoub *et al*., 2019 ^23^	F	64	Flanco	30
Menon *et al*., 2021 ^24^	F	29	Tórax	SD
Presente caso	F	40	Tórax anterior	30

SD: sin datos

Microscópicamente, se observa como una lesión bien definida, generalmente encapsulada, sin aumento del número de células de los capilares o zonas de necrosis, y que se caracteriza por la presencia de estructuras rosetoides con un área central de fibras de colágeno dispuestas en patrón radiado, con células redondas y pequeñas dispuestas en su periferia ^6^^,^^8^^-^^11^.

Se ha intentado clasificar esta neoplasia según el tamaño de las estructuras rosetoides, cuyo diámetro varía generalmente entre 0,1 y 0,65 mm, por lo que se ha propuesto una diferenciación de dichas estructuras en grandes o pequeñas ^12^. Si bien no se tiene precisión sobre el punto de corte para el tamaño que distinga entre estructuras rosetoides grandes y pequeñas, esta clasificación resulta pertinente para el diagnóstico diferencial.

En los casos de schwannomas con estructuras rosetoides grandes, las cuales presentan un centro fibrilar no vascular rodeado de una zona multiestratificada de células redondas y pequeñas, se debe considerar el diagnóstico diferencial con el tumor hialinizante (sic) de rosetas gigantes; mientras que aquellos casos con estructuras rosetoides pequeñas, tan pequeñas que recuerdan en ciertos casos a las rosetas verdaderas de Homer-Wright o de Flexner-Wintersteiner, se debe considerar como diagnóstico diferencial al neuroblastoma ^12^. Si bien puede orientar el diagnóstico diferencial, la utilidad de la diferenciación respecto al tamaño aún no es clara y se necesitan más datos para establecer su aplicación y reproducibilidad ^25^.

Una clave importante en el diagnóstico histológico es que en las áreas adyacentes a estas estructuras rosetoides, se suele observar un patrón más similar al de un schwannoma de patrón convencional, reportado en el 72 al 90 % de los casos. La ausencia de este patrón convencional es la que, de hecho, puede dificultar el diagnóstico histológico; por lo tanto, los estudios de inmunohistoquímica son cruciales para resolver estos casos difíciles ^6^^,^^12^^,^^14^.

 Esta neoplasia comparte las mismas características inmunofenotípicas que se esperan de un schwannoma convencional; además, exhibe una positividad fuerte y difusa para S100, con expresión de marcadores neurales como enolasa neural específica, neurofilamento, PG9.5, SOX10 y proteína ácida fibrilar glial.

Los marcadores para diferenciar el músculo liso o los tejidos fibrohistiocítico, epitelial y vascular, son negativos, con un bajo índice de proliferación de la proteína Ki67.

De los resultados de inmunohistoquímica en los casos evaluados, los marcadores con mejor rendimiento para el diagnóstico de esta neoplasia fueron: S100 (25 de 25, 100 %), enolasa neural específica (6 de 12, 50 %), SOX10 (1 de 1, 100 %), GFAP (5 de 11, 45,45 %), Leu7 (3 de 3, 100 %), neurofilamento (2 de 8, 25 %), β-catenina citoplasmático (1 de 1, 100 %), FXIIIa (1 de 1, 100 %) y vimentina (2 de 3, 66,67 %). Los demás marcadores evaluados resultaron negativos.

En el presente caso, llama la atención la expresión citoplasmática de β-catenina, la cual puede observarse en los tumores de la vaina nerviosa periférica, independientemente de su comportamiento biológico ^26^.

En cuanto a los diagnósticos diferenciales, quizá el más importante es el tumor fusocelular hialinizante (sic) con rosetas gigantes, el cual se considera una variante histológica del sarcoma fibromixoide de bajo grado' ^1^.

Los marcadores de inmunohistoquímica, como MUC4, EMA y CD99, resultan útiles en la distinción de esta neoplasia, así como los estudios moleculares para detectar traslocaciones específicas ^1^^,^^27^. Este diagnóstico diferencial es particularmente importante debido al pronóstico tan diferente que presentan estas dos neoplasias ^2^.

Otro de los diagnósticos diferenciales para tener en cuenta es el neuroblastoma (metastásico a piel y tejidos blandos), el cual podría considerarse cuando las estructuras rosetoides sean pequeñas y recuerden a las verdaderas de rosetas de Homer-Wright, además de tener en cuenta la edad y el sitio de presentación ^28^^,^^29^. En la misma línea, aunque con menor frecuencia, se tienen en cuenta a los tumores de la familia del sarcoma de Ewing, en los cuales podrían aplicarse las mismas consideraciones anteriores, aunque con la adición de los marcadores de inmunohistoquímica, como EWS1, FLI1 y CD99, así como estudios moleculares.

Otro diagnóstico diferencial por considerar podrían ser las lesiones melanocíticas benignas de tipo nevus de Spitz o nevus desmoplásicos. En algunos casos, cuando la población celular sea muy anidada, con nidos pequeños en la dermis, en ausencia de un componente de unión o pigmento melánico asociado, estas lesiones podrían considerarse en el diagnóstico diferencial. Esto se resuelve fácilmente al practicar estudios de inmunohistoquímica, siendo de mucha utilidad los marcadores melanocíticos específicos como MelanA y HMB45 ^12^^,^^20^.

Finalmente, el caso reportado corresponde a una neoplasia con una variante poco frecuente y con escasos reportes en la literatura. Esta variante histológica puede representar un reto diagnóstico en casos específicos, en los cuales es necesario combinar un análisis histológico e inmunofenotípico, para evitar complicaciones relacionadas con el tratamiento y el pronóstico de otras enfermedades consideradas en su diagnóstico diferencial.
